# Circulating P-Selectin Glycoprotein Ligand 1 and P-Selectin Levels in Obstructive Sleep Apnea Patients

**DOI:** 10.1007/s00408-019-00299-0

**Published:** 2020-01-02

**Authors:** P. Horváth, Z. Lázár, G. Gálffy, R. Puskás, L. Kunos, Gy. Losonczy, M. Mészáros, Á. D. Tárnoki, D. L. Tárnoki, A. Bikov

**Affiliations:** 1 Department of Pulmonology, Semmelweis University, Tömő utca 25-29, Budapest, Hungary; 2 Department of Radiology, Semmelweis University, Budapest, Hungary

**Keywords:** Systemic inflammation, Sleep apnea, Comorbidities, Adhesion molecules

## Abstract

**Purpose:**

Obstructive sleep apnea (OSA) is characterized by chronic intermittent hypoxia which induces inflammation in blood vessels leading to the development of cardiovascular comorbidities. Several studies implicated the role of P-selectin in vascular inflammation of OSA. P-selectin glycoprotein ligand 1 (PSGL-1) is the main activator for P-selectin and is involved in immune cell trafficking. However, PSGL-1 has not been analyzed in OSA. The aim of the study was to investigate plasma PSGL-1 and P-selectin levels to have a deeper understanding on their interaction in obstructive sleep apnea.

**Methods:**

Fifty-one untreated patients with OSA and 42 non-OSA controls were recruited. Plasma PSGL-1 levels were determined in evening and morning samples, P-selectin levels were analyzed in morning samples using commercially available ELISA kits. Polysomnography was performed in all participants. OSA was defined by an apnea–hypopnea index ≥ 5/h.

**Results:**

PSGL-1 levels did not differ between controls and OSA patients either in the evening or in the morning. Although, there was no difference between controls (16.9/6.8–40.8 ng/ml) and patients with OSA (19.6/8.4–56.8, *p* = 0.24), patients with severe OSA had increased plasma P-selectin levels (25.6/8.4–56.8 ng/ml) compared to mild OSA patients (14.1/8.5–35.3 ng/ml, *p* = 0.006) and controls (*p* = 0.03).

**Conclusions:**

P-selectin expression relates to disease severity suggesting a pathophysiological role in endothelial cell activation. PSGL-1 levels are unaltered in OSA, suggesting an alternative activation pathway for P-selectin in OSA.

## Introduction

Obstructive sleep apnea (OSA) is characterized by repetitive complete or partial collapse of the upper airways and consequential intermittent hypoxia during sleep. Patients with OSA have a higher risk for hypertension, cardiovascular disease and type-2 diabetes mellitus [1].

Chronic intermittent hypoxia leads to the production of pro-inflammatory mediators, such as interleukin 1 β (IL-1β), IL-6 and tumor necrosis factor-α [2]. These, together with hypoxia itself induce the expression of adhesion molecules, such as P-selectin. P-selectin is expressed upon stimulation by thrombin [3], hypoxia [4] or cytokines [5] by platelets and endothelial cells, however platelet P-selectin expression may be less important in OSA compared to endothelial P-selectin [6, 7]. P-selectin enhances the adhesion between leukocytes and endothelial cells thus slowing down the rolling of leukocytes and facilitating their extravasation. P-selectin has extensively been investigated in OSA [6–12] with some studies reporting elevated blood P-selectin levels [8, 11, 12], but not all [6, 7, 10]. A potential explanation for the contradictory findings could be the presence of comorbid obesity, as one study reported no difference in circulating P-selectin levels between OSA and body mass index (BMI)-matched controls [6]. In addition, blood P-selectin concentrations were related to BMI, but not to the indices of disease severity in another study [8], however this has been contradicted by a further report [10]. Nevertheless, as OSA is frequently associated with obesity [13] which may itself induce systemic inflammation [14], potential effect of obesity has to be taken into consideration when interpreting the data.

P-selectin glycoprotein ligand 1 (PSGL-1) is expressed primarily on the surface of leukocytes [15], but endothelial cells and platelets can also express PSGL-1, albeit in a smaller quantity [16]. Its expression in platelets is induced by thrombin [17], which levels were reported to be increased in OSA [8]. There are no data available if inflammatory cytokine cascade seen in OSA can lead to an increased expression of PSGL-1 in leukocytes. Rather, it seems that inflammation may reconfigure PSGL-1 location on leukocytes to facilitate their binding [18]. In addition, appropriate glycosylation of PSGL-1 is essential for P-selectin binding and optimal leukocyte rolling [18, 16] and glycosylation is increased in active lymphocytes [18]. On the contrary, PSGL-1 is downregulated in apoptotic cells [19]. OSA is characterized by increased levels of pro-apoptotic [7] and decreased concentration of anti-apoptotic [20] molecules. The main receptor for PSGL-1 is P-selectin [21]. The main function for PSGL-1 is to facilitate leukocyte trafficking through endothelial cells. In addition, PSGL-1 has both pro-inflammatory role by inducing IL-8 production [22] and immunoregulatory effects by supporting regulatory T-cell differentiation [16] and inducing the expression of immunosuppressive IL-10 and transforming growth factor-β [23]. The levels of both molecules were found to be reduced in OSA [24]. Although PSGL-1 is an essential molecule for leukocyte differentiation and their trafficking during inflammation, its role in inflammatory diseases is not fully understood [16, 18]. However, PSGL-1 has not been studied in OSA yet.

Sleep fragmentation, another hallmark of OSA leads to an altered production of circulating hormones and cytokines.[7] Investigating evening to morning changes of inflammatory biomarkers may explore acute effects of disturbed sleep. In addition, this holds important methodological considerations, when to collect samples from OSA patients to study inflammatory pathways.

The aim of the study was to analyze the levels of circulating PSGL-1 in OSA. We compared evening to morning PSGL-1 concentrations and correlated with markers of OSA severity and comorbidities. PSGL-1 levels were investigated with its receptor, the P-selectin.

## Materials and Methods

### Subjects and Design

Ninety-three participants were recruited from those patients who were referred to the Sleep Unit of the Department of Pulmonology, Semmelweis University due to suspected OSA (i.e. snoring, witnessed apneas, daytime somnolence). None of the patients had previously been diagnosed with OSA, nor had they been treated with continuous positive airway pressure (CPAP) or mandibular advancement devices (MAD). Exclusion criteria included any uncontrolled chronic disease, history of any malignancy within 10 years, and infection within 2 months. Data for screen failures were not captured.

In the evening, after filling out the Epworth Sleepiness Scale (ESS) and taking medical history, venous blood was taken into EDTA tubes for PSGL-1 measurement. This was followed by an attended full-night polysomnography. The following morning blood pressure was measured, and blood collection was performed in EDTA tubes for plasma P-selectin and PSGL-1 as well as in red top tubes for serum C-reactive protein (CRP), glucose, total cholesterol, high density lipoprotein cholesterol (HDL-C), low density lipoprotein cholesterol (LDL-C) and triglyceride measurements. Blood was sampled at fasting conditions and before taking any medication.

The study was approved by the local Ethics Committee (Semmelweis University, TUKEB 30/2014) and informed consent was obtained from all participating volunteers.

### ELISA Measurements

EDTA-treated blood samples were centrifuged within 2 h at 1500 RPM for 10 min at 4 °C. Immediately following centrifugation, plasma was separated into 250 µL aliquots which were stored at − 80 °C until analysis. Samples were thawed just before the ELISA measurements. PSGL-1 and P-selectin levels were measured using a commercially available ELISA kits (Abcam Human PSGL-1 ELISA kit and Sigma-Aldrich Human P-selectin ELISA kit) according to the manufacturers’ instructions in duplicates. We reported the mean values of the two measurements. We measured PSGL1 levels in samples taken in the evening and in the morning, P-selectin levels were analyzed in the morning samples. Intra assay variability was 6.35% for P-selectin and 25.46% for PSGL-1.

### Polysomnography

Polysomnography was performed as it was described previously [7] using Somnoscreen Plus Tele PSG (Somnomedics GMBH Germany). Electroencephalogram, electrooculogram and electromyogram, thoracic and abdominal respiratory excursions, breath sounds, nasal pressure, electrocardiogram and oxygen saturation were recorded. Sleep stages, movements and cardiopulmonary events were scored manually according to the American Academy of Sleep Medicine (AASM) guideline [25]. Total sleep time (TST), sleep period time (SPT) and minimum oxygen saturation (MinSatO_2_) were recorded. Apnea–hypopnea index (AHI), respiratory disturbance index (RDI), oxygen desaturation index (ODI), arousal index (AI) and total sleep time spent with oxygen saturation below 90% (TST90%) were calculated. An AHI ≥ 5/h was diagnostic for OSA.

### Statistical Analysis

Shapiro-Wilks test was used to test to assess normality which showed non-parametric distribution for P-selectin and PSGL-1 levels. Mann–Whitney *U*-test and Chi-square test were used to compare clinical and demographic characteristics as well as biomarker levels between the OSA and control groups. We applied Kruskal–Wallis test, followed by the Dunn’s test to investigate the differences in biomarker levels among the OSA severity groups. Plasma biomarker levels were correlated with clinical variables using the Spearman’s test. All tests were carried out with R v. 3.1.3. Diagrams were plotted with the ggplot2 package for R. Kruskal Wallis and Dunn’s test were performed with the dunn.test package for R. A sample size of 93 was estimated to find significant differences with an alpha of 0.05, a beta of 0.8 and an effect size of > 0.40 with respect to the asymptotic relative efficiency of non-parametric tests. *p* values < 0.05 were considered significant. Data are presented as mean ± SD or median/range.

## Results

### Patient Characteristics

Following polysomnography, 51 patients were diagnosed with OSA (44 ± 15 years). Sixteen patients had mild OSA (AHI 5–14.9 events/h), 15 had moderate (AHI 15–29.9 events/h) and 20 had severe (AHI > 30 events/h) disorder. Subjects’ characteristics and comparisons between the OSA and control groups are summarized in Table 1.

**Table 1:** Patient characteristics of our study population

	Control *N* = 42	OSA *N* = 51	*p* value
Age (years)	45 ± 16	55 ± 12	< 0.01
Gender (male%)	14	68	< 0.01
BMI (kg/m^2^)	24.33 ± 4.66	31.18 ± 6.20	< 0.01
Hypertension (%)	30	72	0.01
Diabetes (%)	14	21	0.23
Asthma (%)	9	3	0.55
Aspirin use (%)	4.7	17.6	0.11
Clopidogrel use (%)	2.38	3.9	1
Statin use (%)	2	29	< 0.01
Ever-smokers (%)	9	19	0.28
ESS (points)	5.94 ± 3.29	6.76 ± 3.70	0.29
AHI (events/h)	2.1 ± 1.5	31 ± 26.7	< 0.01
RDI (events/h)	11.3 ± 6.4	41.1 ± 26.2	< 0.01
ODI (events/h)	1.21 ± 1.15	28.75 ± 26.92	< 0.01
MinSat (%)	90.72 ± 3.22	79.34 ± 10.08	< 0.01
TST (min)	379.21 ± 64.76	394.6 ± 82.66	0.38
TST90% (min)	0.28 ± 1.37	14.68 ± 21.26	< 0.01
AI (events/h)	45.73 ± 17.27	45.02 ± 24.75	0.89
SPT	416.08 ± 52.74	432.48 ± 70.75	0.27

*AHI* apnea–hypopnea index, *BMI* body mass index, *ESS* Epworth Sleepiness Scale, *RDI* respiratory disturbance index, *ODI* oxygen desaturation index, *TST* total sleep time, *TST90%* total sleep time with saturation under 90%, *AI* arousal index, *SPT* sleep period time

### Circulating PSGL-1 Levels

**Fig. 1:**
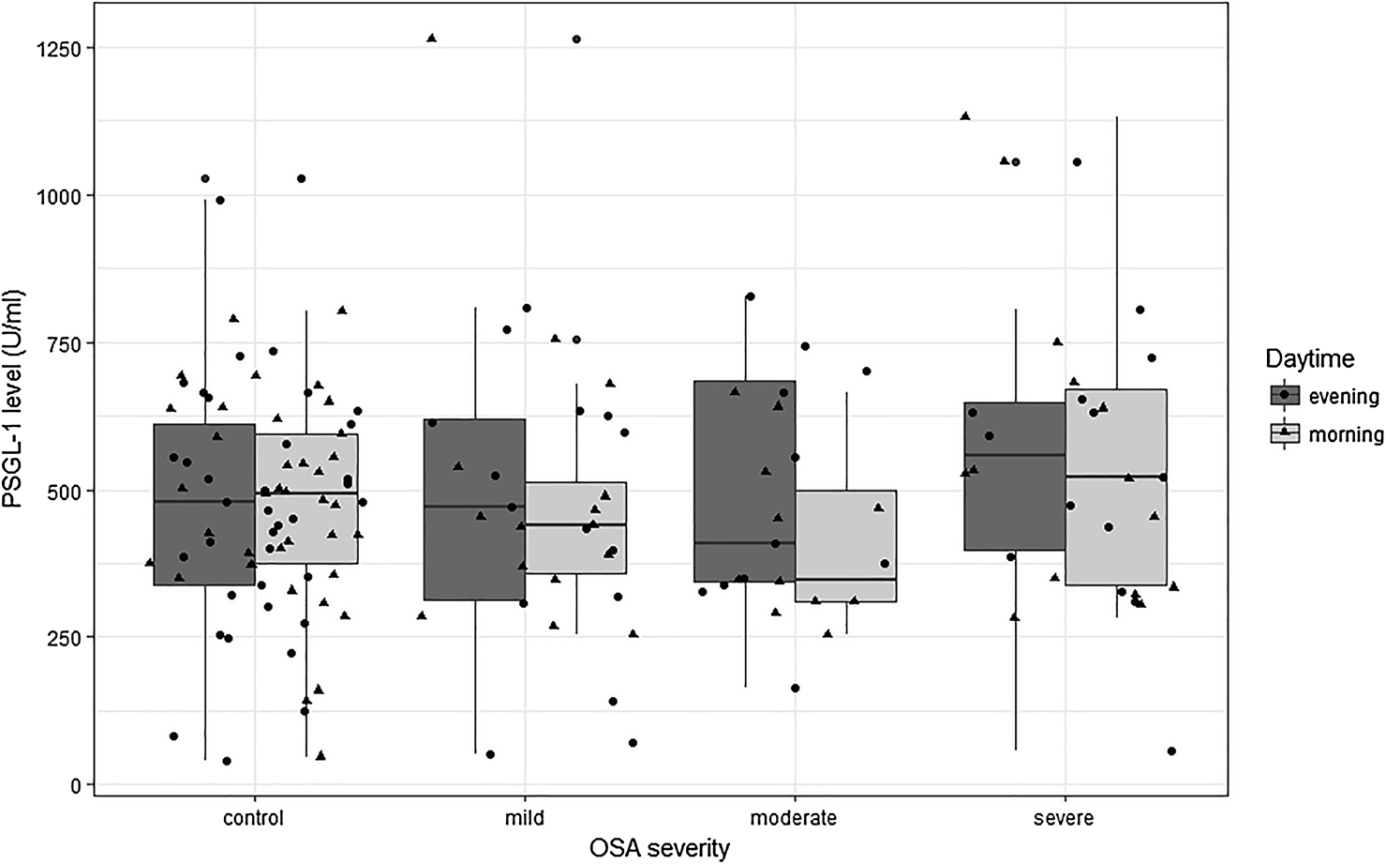
PSGL-1 levels in our population. There was no difference between the OSA severity groups. There was no difference between PSGL-1 levels in the morning and in the evening

PSGL-1 levels did not differ between controls and OSA patients either in the evening or in the morning (494.0/46.9–802.1 U/ml vs. 452.5/254.2–1265.9 U/ml, *p* = 0.67 in the morning, and 480.2/41.3–1027.6 U/ml vs. 497.5/50.2–1054.7 U/ml, *p* = 0.70 in the evening in controls and OSA patients respectively). Similarly, PSGL-1 levels did not change from evening to morning in either group (both *p* > 0.05, Fig. 1.). There was no difference in plasma PSGL-1 levels among the different OSA severity groups at either time point (Table 2). However, there was a significant correlation between the evening and morning PSGL-1 levels (*ρ* = 0.27, *p* = 0.02). There was a trend toward higher PSGL-1 levels in obese OSA patients in the morning (530.7/291.1–1130.8 U/ml vs. 413.4/254.2–1265.2 U/ml, *p* = 0.06) compared to non-obese OSA.

**Table 2:** PSGL-1 and P-selectin levels in the different severity groups. PSGL-1 shows no significant difference, however P-selectin is significantly higher in the severe OSA group compared to controls and mild patients (post-hoc Dunn-test)

	Controls	OSA (mild)	OSA (moderate)	OSA (severe)	*p* value (KW-test)
PSGL-1 (morning) U/ml	494.0 /47.0—802.1/	439.7 /254.2—1265.2/	347.1 /254.7—663.6/	522.0 /283.0—1130.8/	0.43
PSGL-1 (evening) U/ml	480.2 /41.3—1027.7/	470.4 /50.2—808.6/	407.5 /163.8—828.5/	556.4 /55.8—1054.7/	0.78
P-selectin (morning) ng/ml	16.9 /6.8–40.8/	14.1 /8.5—35.3/	17.1 /9.3–48.9/	25.6 /8.4—56.8/	0.01*

*KW-test* Kruskal–Wallis test

*Statistical significance

### Circulating P-selectin levels

**Fig. 2:**
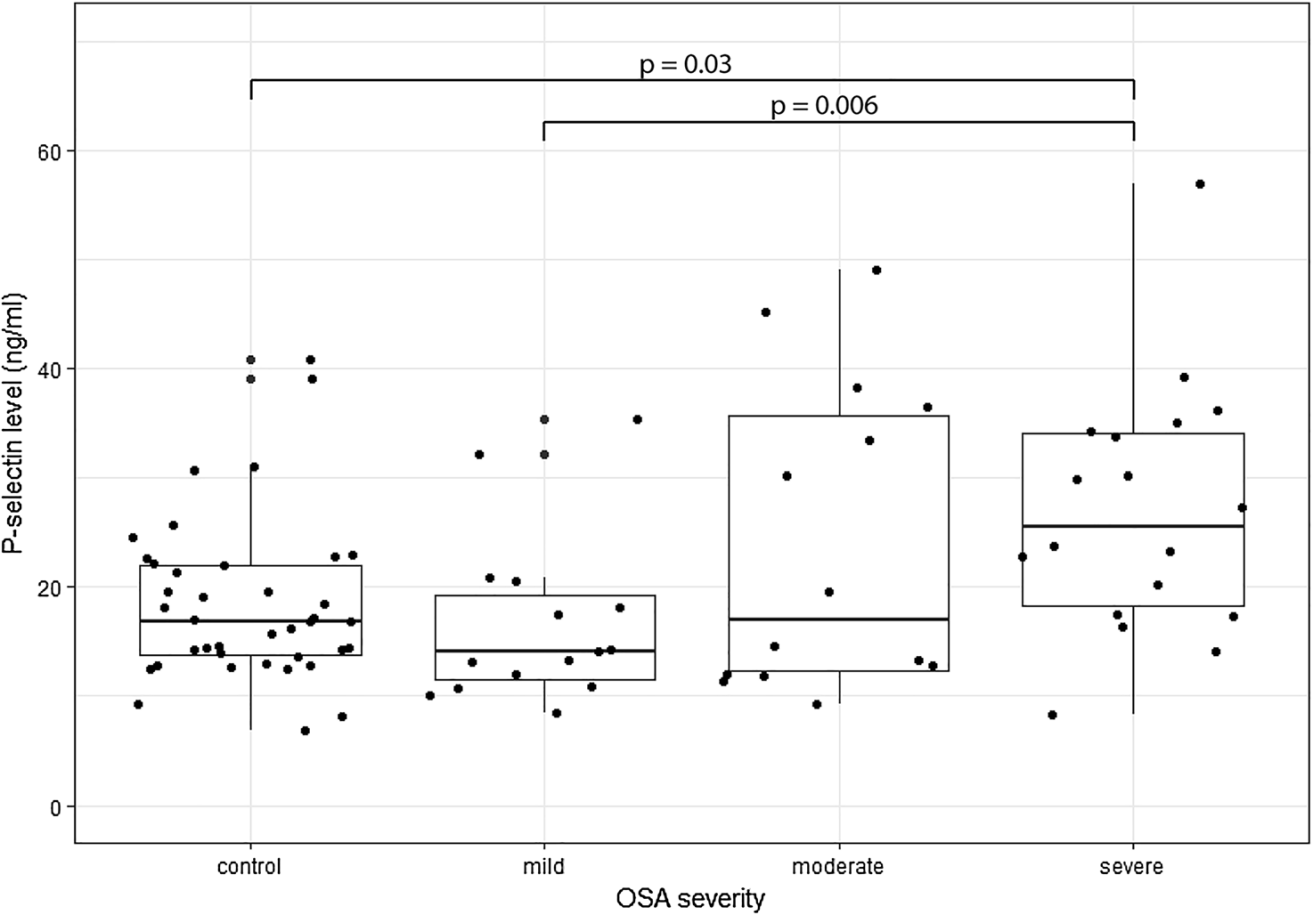
P-selectin levels in our study group. P-selectin level was significantly higher in the severe group compared to controls and mild OSA patients

There was no difference between OSA patients and controls in P-selectin levels (*p* > 0.05). However, circulating P-selectin levels were significantly different among the OSA severity groups (Kruskal–Wallis test, *p* = 0.01, Table 2). After post-hoc testing we found that patients with severe OSA had increased plasma P-selectin levels compared to mild OSA patients (*p* = 0.006) and controls (*p* = 0.03, Fig. 2).

### Circulating P-Selectin Levels and Comorbidities

There was a trend for elevated P-selectin levels in hypertensive patients with OSA (*N* = 37, 22.9/8.4–56.8 ng/ml) compared to patients without hypertension (*N* = 14, 13.7/10.6–35.3 ng/ml, *p* = 0.057). There was no difference between controls with (*N* = 13, 14.5/6.8–40.8 ng/ml) and without (*N* = 29, 18.6/12.6–24.6 ng/ml, *p* = 0.31) hypertension.

P-selectin was significantly higher in obese OSA patients (BMI ≥ 30 kg/m^2^, *N* = 26, 29.9/8.4–56.8/ng/ml) compared to non-obese patients (*N* = 25, 13.7/8.5–38.3 ng/ml, *p* = 0.003). Only two control participants were obese, hence no comparison has been performed in obese and non-obese controls. However, there was no difference in P-selectin between non-obese OSA and non-obese controls (*p* = 0.29).

### Correlation Between Biomarkers and Clinical Parameters

We found no correlation between plasma PSGL-1 levels and any other demographic or clinical parameters, including indices of OSA severity (all *p* > 0.05).

P-selectin levels showed a positive correlation with AHI (*ρ* = 0.24, *p* = 0.02), ODI (*ρ* = 0.29, *p* = 0.005), RDI (*ρ* = 0.29, *p* = 0.005) and BMI (*ρ* = 0.37, *p* < 0.001), systolic and diastolic blood pressure in the morning (*ρ* = 0.30, *p* = 0.005 and *ρ* = 0.34, *p* = 0.001 respectively) and fasting glucose level (*ρ* = 0.38, *p* = 0.001). There was no correlation between plasma P-selectin concentrations and any other investigated variable.

There was no correlation between smoking (either status or pack years) and circulating P-selectin or PSGL-1 levels.

### Correlation Between P-Selectin and PSGL-1 Levels

There was no correlation between P-selectin and PSGL-1 levels either measured in the morning or evening either when all subjects were investigated together, or when patients with OSA or control volunteers were analyzed separately. Analyzing the subgroups, a trend for a moderate correlation between P-selectin and morning PSGL-1 were found in severe OSA patients (*ρ* = − 0.53, *p* = 0.07).

## Discussion

In this study, we investigated the circulating levels of PSGL-1 in OSA together its receptor, P-selectin. We found no difference in PSGL-1 between patients with OSA and control participants, nor was there any overnight change detected. In contrast, there was a significant increase in P-selectin levels in severe OSA patients. However, these results should be interpreted carefully as plasma P-selectin levels were related to obesity apart from OSA severity. Our results suggest that there is no further increase in the expression of PSGL-1 in OSA and enhanced P-selectin in severe disease acts through an unaltered quantity of PSGL-1.

Systemic inflammation plays a role in the development of cardiovascular comorbidities in OSA. Although CPAP is an effective treatment in sleep apnea, it does not acute reduce cardiovascular events in cardiovascular disease [26]. A potential reason could be the fact that CPAP does not change the levels of inflammatory mediators [27]. This emphasizes the need of understanding the elements of systemic inflammation characterizing OSA to identify potential targets for drug development. The current study suggests that PSGL-1 is not altered in OSA, and future studies should investigate different inflammatory pathways.

We measured soluble PSGL-1 which may not necessarily reflect membrane bound PSGL-1 concentrations. It is known that soluble PSGL-1 increases after granulocyte colony-stimulating factor, granulocyte–macrophage colony-stimulating factor, lipopolysaccharide challenge and metalloproteases may cleave membrane bound PSGL-1 as well [16]. The levels of circulating metalloproteases are higher in OSA [28]. The unchanged concentration of soluble PSGL-1 in OSA may be due to balance between events leading to increased cleavage and decreased production of this molecule.

Confirming some previous results [6, 7, 10], we did not find a significant difference in plasma P-selectin levels between OSA and controls. On the contrary, in line with some previous findings [9, 11, 29] severe OSA was associated with higher plasma P-selectin concentrations which were directly related to markers of disease severity (i.e. AHI, ODI and RDI). This is not entirely surprising as mild OSA is associated with the activation of some anti-inflammatory systems [30], suggesting that mild and more severe patients should be investigated separately when analyzing inflammatory mechanisms in OSA. Nevertheless, the relationship between P-selectin levels and disease severity on one hand suggests that enhanced intermittent hypoxia is responsible for increased P-selectin expression. However, on the other hand, P-selectin increase may be due to comorbid obesity, as we found a correlation between P-selectin levels and BMI, and P-selectin levels were higher in obese patients with OSA. Furthermore, there was no difference in P-selectin when non-obese OSA was compared to non-obese controls. OSA is frequently associated with obesity [13] which may itself induce systemic inflammation [14]. Previous studies showed that despite improvement in AHI, neither CPAP [8], nor MAD [12] altered circulating P-selectin levels. This suggests that possibly obesity is a more potent factor maintaining high P-selectin concentrations than intermittent hypoxia. To test this, trials combining CPAP with weight loss programs are required with an aim to investigate P-selectin in OSA. A previous study reporting high P-selectin levels in OSA found a significant association with BMI, but not disease severity [8]. In addition, no difference in platelet P-selectin expression was observed when patients with OSA were compared to weight-matched controls [6]. There was also a tendency for higher plasma P-selectin levels in OSA patients with comorbid hypertension. Interestingly, this difference was not present in non-OSA subjects. In addition, P-selectin levels were related to blood pressure results. This emphasizes that P-selectin may play a role in hypertension and subsequent atherosclerosis characterizing OSA [31].

Sleep fragmentation may induce systemic inflammation [32]. However, neither PSGL-1, nor P-selectin levels were influenced by sleep quality indices. This is in line with a previous study reporting no differences in P-selectin levels between sleepy and non-sleepy patients with OSA [10]. There was no correlation between these molecules and Epworth sleepiness score, nor PSGL-1 levels changed after sleep. Our results suggest that sleep does not influence the levels of PSGL-1 itself.

A potential limitation of the study that the controls and patients with OSA were not matched. Patients with OSA were older, had higher BMI, had higher prevalence of males, hypertension and statin use. This is not surprising as these factors are associated with OSA [13]. Most notably, there was a significant association between plasma P-selectin levels and hypertension as well as BMI. Therefore, increased levels observed in severe OSA must be interpreted carefully. Of note, neither P-selectin, nor PSGL-1 concentrations were related to age, gender or medication usage, still because the current study was not powered to investigate these relationships, future studies should aim for matching OSA and control groups.

In summary, neither P-selectin nor PSGL-1 levels were altered in OSA. However, unlike PSGL-1, P-selectin concentrations were increased in severe OSA suggesting that either intermittent hypoxia or more probably comorbid obesity lead to endothelial and/or platelet activation expressing P-selectin. However, unchanged PSGL-1 does not rule out the increased activation of P-selectin on the endothelium in severe OSA and consequently, the accelerated extravasation of leukocytes as functional changes in PSGL-1 might facilitate ligand-receptor binding.
